# Go.Data as a digital tool for case investigation and contact tracing in the context of COVID-19: a mixed-methods study

**DOI:** 10.1186/s12889-023-16120-w

**Published:** 2023-09-04

**Authors:** Sara Hollis, Jeni Stolow, Melissa Rosenthal, Silvia Edith Morreale, Lina Moses

**Affiliations:** 1 Health Emergencies Programme, World Health Organization, Geneva, Switzerland; 2 School of Public Health and Tropical Medicine, Tulane University, New Orleans, LA USA

**Keywords:** Contact tracing, Coronavirus, COVID-19, Technology, Mixed methods, Digital health, mHealth, Outbreak response

## Abstract

**Background:**

A manual approach to case investigation and contact tracing can introduce delays in response and challenges for field teams. Go.Data, an outbreak response tool developed by the World Health Organization (WHO) in collaboration with the Global Outbreak Alert and Response Network, streamlines data collection and analysis during outbreaks. This study aimed to characterize Go.Data use during COVID-19, elicit shared benefits and challenges, and highlight key opportunities for enhancement.

**Methods:**

This study utilized mixed methods through qualitative interviews and a quantitative survey with Go.Data implementors on their experiences during COVID-19. Survey data was analyzed for basic univariate statistics. Interview data were coded using deductive and inductive reasoning and thematic analysis of categories. Overarching themes were triangulated with survey data to clarify key findings.

**Results:**

From April to June 2022, the research team conducted 33 interviews and collected 41 survey responses. Participants were distributed across all six WHO regions and 28 countries. While most implementations represented government actors at national or subnational levels, additional inputs were collected from United Nations agencies and universities. Results highlighted WHO endorsement, accessibility, adaptability, and flexible support modalities as main enabling factors. Formalization and standardization of data systems and people processes to prepare for future outbreaks were a welcomed byproduct of implementation, as 76% used paper-based reporting prior and benefited from increased coordination around a shared platform. Several challenges surfaced, including shortage of the appropriate personnel and skill-mix within teams to ensure smooth implementation. Among opportunities for enhancements were improved product documentation and features to improve usability with large data volumes.

**Conclusions:**

This study was the first to provide a comprehensive picture of Go.Data implementations during COVID-19 and what joint lessons could be learned. It ultimately demonstrated that Go.Data was a useful complement to responses across diverse contexts, and helped set a reproducible foundation for future outbreaks. Concerted preparedness efforts across the domains of workforce composition, data architecture and political sensitization should be prioritized as key ingredients for future Go.Data implementations. While major developments in Go.Data functionality have addressed some key gaps highlighted during the pandemic, continued dialogue between WHO and implementors, including cross-country experience sharing, is needed ensure the tool is reactive to evolving user needs.

## Background

**Fig. 1:**
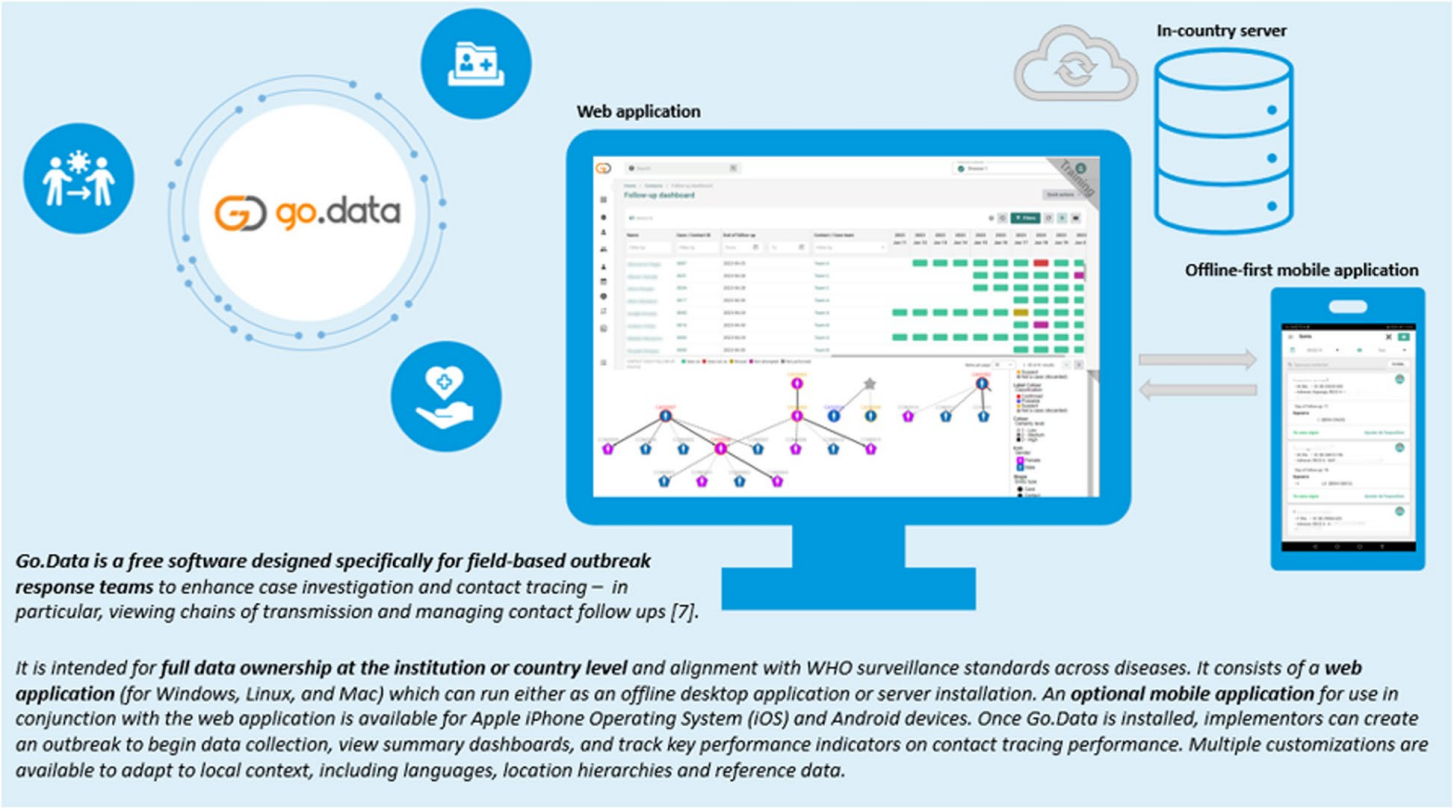
Brief overview of the Go.Data tool

Responding to infectious disease outbreaks requires careful management of large amounts of data on cases, their contacts, and exposure events. In many settings, a manual and largely paper-based approach to case investigation and contact tracing has introduced strains on field teams and delays in response [1–4]. To respond to Member States’ requests for a free, flexible, and open-access platform designed specifically for the outbreak context, the World Health Organization (WHO) and Global Outbreak Alert and Response Network (GOARN) partners hosted a workshop in 2016 to collaboratively outline key functionalities for field data collection tools [5, 6]. Outputs from this workshop, together with years of collective experience across GOARN deployments, formed the basis for key requirements and principles underpinning the subsequent Go.Data development (Fig. 1).

The first version of Go.Data was released in 2019 and deployed later that year in response to an outbreak of diphtheria in Cox’s Bazar, Bangladesh followed by responses in Kasese, Uganda and North Kivu, Democratic Republic of the Congo for the 2018–2020 Ebola outbreak [8, 9].

Prior to COVID-19, a range of digital tools were implemented during outbreaks, including for contact tracing [10–19]. Interest in the role that digital health can play within public health response has only increased during the pandemic, where contact tracing was widely adopted across countries, exponential caseloads overwhelmed traditional information management systems, and long-term national underinvestment in digital reporting infrastructure was brought to the forefront [20–23].

In the case of Go.Data, the tool saw a rapid surge in demand during the COVID-19 pandemic – from previous use within smaller scale outbreaks to implementations in over 65 countries or territories and 115 institutions by the end of 2021 [24, 25]. Despite its widespread use, there was limited documentation of Go.Data implementations across contexts, and key learnings remained largely anecdotal in nature through informal exchanges with implementors via WHO consultations or technical meetings [26–28].

The goal of this mixed methods study was to characterize the landscape of Go.Data implementations during COVID-19, identify enabling factors and impacts of Go.Data on response efforts, and highlight common challenges and areas where future enhancements are needed.

## Methods

### Study design and setting

A mixed methods study was conducted between April and June 2022 with global recruitment and participation across all WHO regions. The study utilized both quantitative and qualitative methods through a pre-interview quantitative survey and qualitative in-depth interviews with Go.Data users. A mixed methods design was chosen to obtain a full range of stakeholder views and distill key themes across Go.Data implementations. Interviews elicited transparent and nuanced feedback on successes, challenges, and lessons learned, while pre-interview surveys captured complementary quantitative descriptors such as institutional and participant profiles, scope of implementation, and logistics of Go.Data roll-out.

### Study participants and recruitment

Study participants were primary focal persons, defined as the main project lead during the time of initial roll-out, across all eligible implementations where Go.Data was used during the COVID-19 response. The research team screened the WHO-Go.Data implementation database to identify eligible Go.Data implementations and their corresponding focal person. An implementation was deemed eligible if it was successfully installed *and* used by response personnel between January 2020 and April 2022. Where corresponding personal contact information was missing, WHO regional and country office teams followed up with in-country counterparts for additional verification. The resulting list of individuals were invited to participate, or to otherwise identify a more suitable representative from their institution. All participants signed informed consent and due diligence forms. Sampling for interviews continued past saturation, to ensure appropriate regional representation where possible. This entailed additional contact to WHO regions without existing representation in request of interview participation. Recruitment stopped once there was representation from all six WHO regions. Prior to each interview, participants were asked to fill out an online pre-interview survey (Qualtrics, Provo, UT).

### Data collection and analysis methods

The research team comprised of an external GOARN evaluation team (LM, JS, MR) and WHO personnel supporting the Go.Data project (SH, SM), all of which were collectively trained on the study protocol, qualitative methods, and interviewing techniques. Before initiating data collection activities, the research team jointly reviewed and piloted the interview guide and survey to establish validity and reliability.

The interview guide contained open-ended questions probing interviewees to discuss topics such as the appeal of Go.Data, their experiences installing and implementing the tool, and their perceived successes and challenges working with the software during the COVID-19 response. The languages used for interviewing were English, Spanish, and French and were adapted at the request of the interviewee. Interviews (ranging from 20 to 70 min) were conducted virtually via Zoom and were recorded and automatically transcribed with consent from the interviewees. All interview transcripts were reviewed by the research team to verify content. Non-English transcripts were sent to a professional translation and transcription services prior to analysis, and all finalized transcripts were organized, stored, and analyzed in NVivo 12 software to allow for memoing, coding, and categorizing of interview responses. The qualitative codebook went through several rounds of iterative review by the research team until consensus on the final coding frame was reached. All interviews were dual coded and reviewed as per the 10% minimum set forth for inter-rater reliability [29, 30]. The qualitative line-by-line coding included inductive as well as deductive analysis. Deductive reasoning was used to compare qualitative input to survey responses. Inductive reasoning was used to look beyond the pre-determined questions to see what alternative patterns emerged.

In contrast to the open-ended structure of the qualitative interview, the quantitative survey aimed to collect close-ended data pertaining to the logistics of using Go.Data, for example, the size of implementation teams, the type of institutions using Go.Data, prior tools used for contact tracing, the length of time it took users to install and implement the software, and in what settings the Go.Data tool was used. Participants were asked to complete this survey prior to the scheduled time of the interview and to pass along the survey to other co-implementers from their institution who may be able to best answer the survey questions.

In alignment with mixed methods best practices, the qualitative interview guide and quantitative survey instrument echoed core questions for the purpose of triangulation [31]. Qualitative findings were compared to the quantitative results and iteratively discussed within the research team until a consensus was reached. Finally, two rounds of member-checking, the process of garnering feedback from study participants prior to dissemination, took place to ensure key findings resonated and were representative of participant experiences [32]. The first round of member-checking took place with the Go.Data team and the second round of member-checking occurred with all study participants, via email correspondences requesting any clarifying comments and reflections. The results presented in this document reflect the findings and feedback from all rounds of analysis and member-checking.

## Results

### Study sample characteristics

In April 2022, the research team reached out to 95 focal persons associated with an eligible Go.Data implementation. After initial correspondence and triangulation of implementation details, 15 among the 95 were excluded where Go.Data was eventually not implemented or the focal point could not be identified. After two follow-up reminders, a total of 33 primary focal persons agreed to participate and the rest were classified as no response (*n* = 47). The study team conducted interviews across English (*n* = 27), French (*n* = 4), and Spanish (*n* = 2) languages. In total, 41 corresponding pre-interview surveys were received. Slightly more surveys were collected than interviews as some implementation teams had more than one focal person or key Go.Data team members that opted to fill a survey to provide feedback. After coding of qualitative interview data, 28 codes were identified and triangulated with quantitative data across key categories aligned with study aims.

**Fig. 2:**
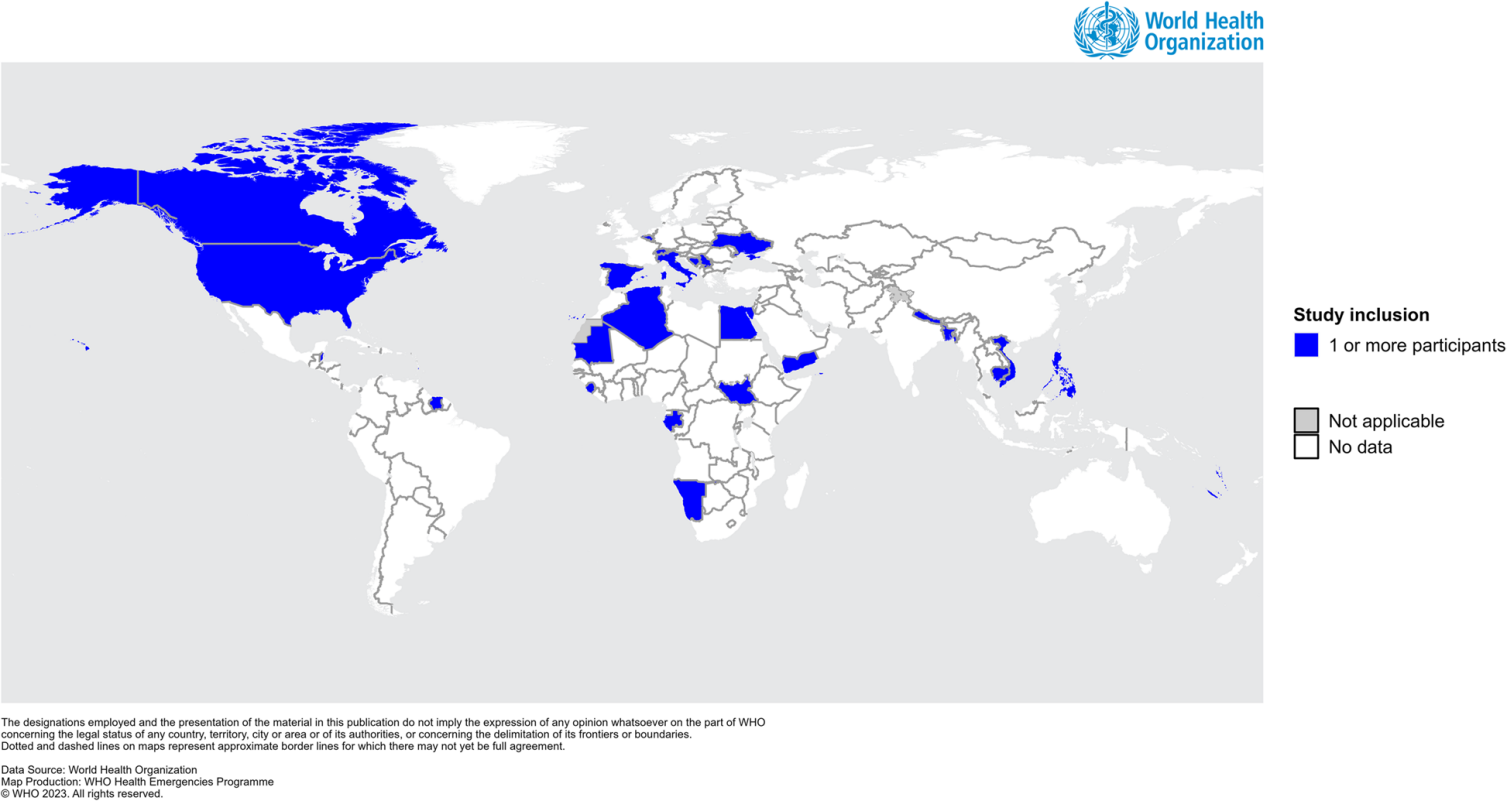
Geographic representation of study participants

Interviewees and survey respondents reflected representation across all six WHO regions and 28 countries (Fig. 2) and acted in variety of roles at their institution, both in general and for the COVID-19 response (Table 1). Of note, there was a significant discrepancy between functions supported within the response and “fixed” roles at the institution during peace-time – for example, an overwhelming majority of respondents supported the response in information technology (IT) (80%) or supervision (78%) capacities, while roles typically requiring these skills, such as IT specialists or contact tracing coordinators, represented only 7% and 10% of fixed roles, respectively.

**Table 1:** Participant characteristics

**Characteristics**	Total eligible implementations (*n* = 95) n (%)	Interviews (*n* = 33) n (%)	Surveys (*n* = 41) n (%)
**WHO Region**			
Africa	10 (11)	6 (18)	5 (12)
Americas	24 (25)	6 (18)	4 (8)
Eastern Mediterranean	4 (4)	2 (6)	3 (7)
Europe	26 (27)	10 (30)	16 (39)
South-East Asia	7 (7)	3 (9)	6 (14)
Western Pacific	24 (25)	6 (18)	7 (17)
**Primary role at institution**			
Data/information management officer			15 (37)
Other			8 (20)
Epidemiologist			6 (15)
Contact tracing coordinator			4 (10)
Clinical/medical officer			3 (7)
Information technology specialist			3 (7)
Analyst/biostatistician			2 (2)
**Role(s) within COVID-19 outbreak response**			
Information techology support			33 (80)
Supervision/administration			32 (78)
Training/capacity building			27 (66)
Epidemiology/surveillance			26 (63)
Other			13 (32)
**Still part of the Go.Data team within institution**			
Yes			34 (83)
No			7 (17)

### Aim 1: Landscape of Go.Data implementations

The first objective of this study aimed to document and characterize the landscape of Go.Data implementations during COVID-19. Notably, the distribution of study participants across all six WHO regions roughly mirrored the regional distribution of eligible Go.Data implementations during COVID-19 (Table 1), and information on the scope and type of implementations is shown in Table 2. The majority of participating implementations (58%) were conducted by relevant governmental bodies such as the Ministry of Health or national public health institute and entailed national scope (45%) or focus on a particular subnational area (42%). While most implementations introduced Go.Data as a practical tool to support case listing, contact listing and contact follow-up (71%), several others positioned Go.Data for research purposes that entailed detailed case-level data collection on specific sub-groups (10%) or only a storage of cases and contacts (10%). Interviews further clarified that global research initiatives such as WHO Unity Studies faciliated Go.Data use as a data collection instrument for the First Few X (FFX) cases and contact investigation protocol and assessment of risk factors for COVID-19 in health workers [33]. Among interviewees not using Go.Data for contact follow-up, some mentioned this was due to the immense data volumes and data entry burden as contact tracing scaled.

**Table 2:** Go.Data implementation characteristics

Characteristics (*n* = 31)^a^	n (%)
**Planned implementation scope**	
National	14 (45)
Subnational	13 (42)
Institutional^b^	4 (13)
**Institution Type**	
Government (Ministry of Health, or Other) or public health institute^c^	18 (58)
United Nationsor other multilateral agency	10 (32)
University or research Center	2 (6)
Nongovernmental organization	1 (3)
	-
**Primary planned use for Go.Data**	
Case listing, contact listing and contact follow-up	22 (71)
Research and protocols requiring detailed data collection on cases	4 (13)
Case listing and contact listing (no follow-up)	3 (10)
Data visualizations only	2 (6)

^a^collected from survey responses and de-duplicated by institution. Two in-depth interviews did not have a corresponding survey response

^b^roll-out within individual institutions, such as hospitals, universities or research centers

^c^including local public health authorities/governmental bodies

### Aim 2: enabling factors and positive impact of Go.Data

The second objective of this study aimed to identify the enabling factors and perceived positive impacts of Go.Data on COVID-19 response efforts. Among survey responses, the elements appearing most frequently were WHO endorsement (85%), specific features of interest (61%), free cost (61%), and ease of implementation (59%). Features regularly cited in interviews related to data visualization and chains of transmission, as evidenced in the below quote:

*“Focusing on those very nice features which attracted us to it in the first place, like the graphical description, depiction of the chains of transmission and the mapping and so on, because those are the things which really attracts you to it.”* *(Interviewee 29)*

Given that several factors independent from tool functionality arose in survey responses, these concepts where explored further in the in-depth interviews and are outlined below.

#### WHO endorsement

WHO endorsement was a recurring theme across diverse institutional contexts. Interviewees emphasized that Go.Data being a WHO tool not only increased their trust in the software but facilitated buy-in from key stakeholders, such as Ministries of Health, financing bodies and other public health agencies – often necessary for obtaining initial approvals to proceed. As seen in the next quote, interviewees expressed the risk involved in the uptake of the software and how WHO endorsement helped to alleviate this:

“*…it was a risky time to try something now…but the WHO logo felt reliable and helped us convince our stakeholders that...downloading the software was the right choice to make during the unknown time of the pandemic.” (Interviewee 14)*

Aside from trust and reliability, interviewees alluded to benefits of accessing the wider WHO network. Many interviewees cited the opportunities for cross-country exchange and bi-directional dialogue with the WHO team, on Go.Data and contact tracing more broadly, as a key element in their continued use of the tool. Existing WHO Member State forums or connections facilitated through WHO country office presence facilitated these opportunities, in the eyes of interviewees:

*“….having ample experience from other countries to pull in and show was also one [benefit]… we had a number of exchange with [other country teams], because they had implemented at scale…we organized a couple of discussions with them and are even planning to go visit [the country] with a small team from the Ministry of Health.” (Interviewee 19)*

#### Accessibility and adaptability

More than half of survey respondents (59%) and all interviewees mentioned Go.Data’s free cost as an important factor in selecting the tool. Notably, interviewees described that this ensured constrained budgets could be directed towards the necessary human resources and IT infrastructure for adapting the Go.Data tool to the their surveillance context:

*“Go.Data was available, it was off the shelf, and it was free...it’s an important consideration, even though we're a large organization. But free means one thing… there was a lot of work making it work within our context, with which was an investment.” (Interviewee 23)*

Many interviewees expanded on the theme of adaptability, noting that flexible and ready-to-use components enabled rapid tailoring to local needs – for example, language tokens to quickly translate the user interface, default disease outbreak templates aligned to WHO standards for minimum variables, and custom questionnaire builders to align with national investigation forms. These were seen as greatly expediting the set-up process, ensuring quality was upheld, and creating a structure for future outbreaks, as evidenced in the following quotes:

*“So, I think the easiest [thing] about Go.Data is that it's easy to install. You know just one click and it will be set up in an easy to understand all the variables... With Go.Data, you can easily add or remove whatever you want.” (Interviewee 3)*

*“...from our side, you can plan to use the system for other outbreaks that are not COVID. Even when COVID ends, for other outbreaks we can continue to use the system.” (Interviewee 8)*

#### Flexible support modalities

While a few institutions simply downloaded Go.Data and proceeded with implementation self-sufficiently, most survey respondents (85%) had sought out at least one mechanism of Go.Data support prior to or during roll-out. This was largely through online modalities, such as virtual training sessions with the Go.Data team (71%), online user manuals (68%) and the self-paced OpenWHO training course (62%). Although only 29% of survey respondents made regular use of the Go.Data Community of Practice, those who did cited its value during interviews for both troubleshooting urgent IT issues and connecting informally with users from different contexts. When asked about their team’s experiences engaging with support functions, one interviewee stated:

*“I'd like to thank the Go.Data team for their support. For their understanding about [our] challenges, and [how they] adapt[ed] our comments so far. I hope you can continue this journey and improve the system…now that we have developed a digital health strategy where everything will be digitalized and then interconnected.”* *(Interviewee 4)*

#### Go.Data impact on COVID-19 response efforts

Key benefits of Go.Data implementation were summarized by survey respondents (Table 3) and further explored in interviews. Participants echoed the perceived benefit that Go.Data brought to daily response activities in terms of structure and standardization across data and people processes.

**Table 3:** Go.Data’s impact on COVID-19 response efforts

Select which of the following aspects of your COVID-19 response were enhanced, after Go.Data introduction (n = 38)^a^	n (%)
Standardization and cleanliness of data	28 (70)
Structure to contact listing and follow-up	28 (70)
Timeliness of data	26 (65)
Reporting and indicators	23 (57)
Team and task organization	18 (45)
Real time chains of transmission visualization	17 (42)
Contact tracing follow-up performance	15 (38)
Mechanism for learning more about contact tracing	15 (38)
Linking IDs of cases with lab data	10 (25)
None of these	3 (8)
Other	3 (8)

^a^from survey respondents; multiple responses allowed; 3 provided no response

Surveys and interviews noted that not all institutions had a contact tracing data system in place, nor a dedicated multidisciplinary team to perform activities, prior to COVID-19 and Go.Data introduction. Of those institutions who had some infrastructure in place, most managed all related data on paper forms, Google Forms, or shared Excel files prior to Go.Data implementation (Table 4). Many re-iterated that even if basic, prior infrastructure provided a starting place for Go.Data configuration and task organization to begin.

**Table 4:** Data management strategies prior to Go.Data introduction

What strategy was generally used for managing your case investigation and contact tracing data prior to Go.Data introduction? (*n* = 38)^a^	n (%)
Paper forms, later input into excel or other web-based tool	31 (76)
Direct entry into excel or other web-based tool	13 (32)
Paper forms only	9 (22)
Other (specify)	2 (5)

^a^from survey respondents; 3 provided no response

In this way, introducing Go.Data was cited by many interviewees as means to consolidate and formalize workflows, if and where they existed, and establish these as a foundation for future outbreaks. This meant complementing existing teams and ensuring staff across pillars could input data simultaneously into one system, as evidenced by the two quotes below:

*“…our [previous system] created a lot of confusion, because we had different data on various excel sheets. So for us, we were looking for one true tool—a one stop shop where everybody can enter data…[someone] recommended Go.Data, and realized that that is just what we needed.” (Interviewee 2)*

*“…[Go.Data] was a way to centralize all the data coming in so quickly. We didn’t have a system in place before Go.Data to handle the load coming in…we also didn’t know how massive COVID [would be]…” (Interviewee 3)*

Aside from data management alone, improvements in data analysis tasks were cited across surveys and interviews alike. Several interviewees noted increased efficiency in producing and communicating epidemiological information for decision makers through out-of-the-box analytics features:

*“...[Go.Data] made it easy...to index and source and build out visualizations and graphs...heat maps and other things... [and to] have a system that's functional and not overly complicated…”* *(Interviewee 22)*

Importantly, all participants emphasized the value-add of being better prepared for future outbreaks after Go.Data implementation and the wisdom of such advice as *“…don't try to implement something new in times of crisis”* (Interviewee 10). Across survey respondents, already 12% noted having expanded Go.Data platform for other outbreaks beyond COVID-19, and interviewees echoed similar sentiments:

*“Yes we are currently using Go.Data for the outbreak responses, one is for diptheria and the other’s for COVID…and it is the initial part [of] actually why we started this Go.Data implementation.” (Interviewee 7)*

### Aim 3: challenges and opportunities for enhancement

The third objective of this study aimed to identify users’ perceived challenges during Go.Data roll-out and which enhancements and support should be prioritized. There were several specific challenges noted by survey respondents, many of which were IT-related, but some of which implied financial and workforce constraints, as shown in Table 5.

**Table 5:** Challenges experienced during Go.Data implementation

Select which challenges you experienced during implementation (*n* = 36)^a^	n (%)
Problems during platform updates	22 (56)
Difficulties managing load on server / optimizing server appropriately	18 (46)
Connectivity	12 (31)
Insufficient IT support	12 (31)
Turnover of contact tracers/staff	12 (31)
Installation	9 (23)
Budget constraints	8 (21)
Other	3 (8)

^a^from survey respondents; multiple responses allowed; 5 provided no response

Some challenges were reported regardless of institution type and scope, such as problems with installation, platform updates and server load across national, subnational and institutional implementations alike. However, certain resource challenges such as turnover of staff and budget constraints were only cited by national and subnational implementations. Interviews elucidated a more nuanced understanding of common challenges and how they could potentially be addressed, outlined in the following sections.

#### Personnel and skill-mix within implementation teams

Many interviewees noted that while some IT tasks during installation or configuration could be tedious or introduce unforeseen errors if small details were overlooked, many technical issues that persisted, or were ultimately addressed, depended on the capacity and skill-mix of the team. This included, for example, having at least one IT specialist that could set up the server, monitor platform performance over time, and report any issues rapidly to the Go.Data IT support team. Survey data showed that 37% of teams needed to eventually hire contractors to assist with Go.Data setup and use, reflecting that there were varying degrees of IT capacity and existing infrastructure in many institutional settings. One interviewee reflected this sentiment, describing that a diverse baseline level of IT capacities can introduce challenges for supporting a tool’s roll-out globally:

*“…Go.Data tries to be all things to all men in the sense of trying to meet the needs of a diverse range of countries….[who] may not have the same level of networking or compatible [IT] facilities.” (Interviewee 31)*

Some interviewees noted that during major upgrades, teams under time pressure had little time for extensive application programming interface (API) testing or scanning dense documentation. Several recommended more predictable communications from the Go.Data team around IT bugs and fixes and clearer software release notes.

Beyond limitations in IT capacity, participants also reported the lack of managerial staff to oversee contact tracers, in line with findings in Table 1. Per interview data, most teams had fewer than five people in managerial roles and up to hundreds of contact tracers, data collectors or laboratory staff. Training teams on Go.Data at such scale was challenging, and 12.2% of survey respondents reporting no structured and cohesive training that accompanied Go.Data implementation. Furthermore, interview data revealed the difficulty in continuously training personnel despite COVID-19 risk reduction policies which prevented large gatherings and frequent staff turnover.

#### Data volume

Almost all interviewees described the immense challenge of increasing data volumes that the COVID-19 pandemic yielded. This became overwhelming for users, especially as early 2020 versions of Go.Data were reportedly less performant in visualizing large amounts of data:

*“The problem is there are some limitations in Go.Data which we cannot configure to be more user friendly…especially when it comes to big data, because I know at the beginning [it was created] for smaller outbreaks, but when it comes to COVID, especially right now we are using [Go.Data] for lab results management as well, with two million records. So, we face some challenges in terms of speed, in terms of data visualizations, especially with analyzing.. there are some limitations in terms of that, so we needed to export to a third-party program.” (Interviewee 11)*

Interviewees recommended potential ways to improve usability with large datasets, such as improved pagination and advanced filter functionalities when locating cases and contacts of interest:

*“… [in a search] I can only see the maximum is 50 on a page when you have 800 or thousand. Exactly so you're looking at one, and when you want to go back you want to go back to the same search it brings you back to zero, so you have to start the search again over and over and over so it’s impossible.” (Interviewee 6)*

Noted as less urgent, but frequently mentioned, were aspects of the platform deemed too rigid, such as the inability to add or modify core variables in the case and contact module. Additionally, the lack of localization (not adjusting to a user’s specific time-zone of interest) when records were created or modified combined with few recognizable features that most medical professions were familiar with in other digital systems (e.g., comment boxes and electronic signatures) were highlighted across some interviews. These issues in particular posed significant roadblocks for implementations where Go.Data was used by clinical staff, as highlighted in the following quote:

*“The lack of a timestamp is a major legal issue. If it's 10:27 now I should have legally a timestamp 10:27 when I'm entering my note. It’s all legal matters because I can go in and add a whole bunch of things in a certain area and the next person can come and delete it there's no way to backtrack that legally if you go to court. You have nothing to stand on.” (Interviewee 17)*

#### Data entry burden

Not mentioned in the survey but seen throughout the interview data was the challenge of data entry burden on staff with limited bandwidth. Several interviewees mentioned that it was more common for medical professionals tasked as contact tracers during COVID-19 to push back on Go.Data use, as it was seen as an additional burden during an already chaotic and strenuous time for the medical community.

*“There are some people who still reason that data is for techies not for the doctors...so we had a bit of culture gap to bridge...this idea that ‘this is a crisis I need to be a doctor providing care’ ...that data inputting was almost bad practice because it was a crisis and it was inappropriate to ask them to do that.” (Interviewee 29)*

#### Institutional approvals

Across surveys and interviews, bottlenecks to timely implementation were discussed, with key intervals such as time to approval and time to installation discussed. Timelines varied across institutions due to factors such as the level of previous training and sensitization on Go.Data, planned implementation scope, size of the Go.Data implementation team and political context. Although a portion of survey respondents (20%) reported receiving institutional or governmental approval, if needed, within 72 hours, nearly half (47%) waited longer than one month to obtain approvals. The main reason for approval delay was related to stakeholder skepticism to try a new software on national servers during a crisis such as the COVID-19 pandemic, as illustrated by interviewee 24:

*“The Ministry of Health was unsure at first…and took a long time to talk it over…it was a big and new software that needed access to our national [secured] servers during a time where people were already untrusting of the government…so you can see…it took some time to deliberate.” (Interviewee 24)*

Once proper approvals were obtained, participants noted that installation and configuration itself was relatively rapid, with nearly half (42%) completing installation within 72 hours and 21% within 24 hours.

## Discussion

This study highlighted the overall potential for Go.Data to enhance case investigation and contact tracing activities across diverse contexts, both during the COVID-19 pandemic and for future outbreaks. It also elicited common enabling factors and issues encountered during implementation and scale. Importantly, it crystallized the challenges inherent in implementing a new information system during a large-scale emergency, where considerable constraints on workforce and system capacities can minimize effectiveness of surveillance activities, regardless of the tool used [34, 35].

Factors such as WHO endorsement, accessibility, adaptability, and flexible support were important considerations for implementors during the tool selection phase. Maintaining accessibility should remain central to the Go.Data project ethos to minimize bespoke outsourcing for specific personnel in future responses. Go.Data’s use during COVID-19 demonstrated the tool’s applicability both in high-income and low-income settings alike [24, 25, 36], but some required significantly more implementation support than others. Given this reality, decentralization of project support at the WHO regional and country levels can help further ensure that quality and coverage of support is maintained, while ensuring online support materials are frequently updated and available in multiple languages. The ability for the Go.Data project to initiate cross country exchanges through WHO and GOARN forums is a valuable aspect of implementation that was echoed across participants overall. These forums, in addition to the Go.Data Community of Practice, should be further leveraged as the tool evolves to ensure it remains fit-for-purpose and is increasingly country owned, particularly as efforts towards a fully open-source software license through the WHO’s Open Source Programme Office are realized [37–39].

Study findings suggested Go.Data’s inherent compliance with WHO surveillance standards across diseases was seen as credible by public health responders worldwide, thereby creating efficiencies in COVID-19 response teams. Open-access and standards-based toolkits for public health practitioners are becoming increasingly important to both ensure alignment with data management and analysis best practices and de-duplicate efforts [4, 40, 41]. Given the challenges posed by introducing new standards (among other technical or logistical hurdles) during an emergency, study findings reiterated that a platform like Go.Data is most optimally introduced during the preparedness phase. This allows for ample sensitization across high-level stakeholders and end users, alike, and contributes to ensuring data streams we are building today can solve for tomorrow’s questions [42]. Embedding tools of interest into existing curriculums with global reach, such as Field Epidemiology Training Programs (FETP), holds great potential for future Go.Data implementation activities, given the critical role of FETPs in the global workforce to rapidly detect and respond to outbreaks [43]. Such actions could ensure that systems are in place at national, local, and institutional levels and can be scaled as needed while modernizing the toolbox of field epidemiology cohorts over time.

Although surveillance staff and data managers are the most obvious users of the Go.Data tool, other key members played crucial roles throughout interviewee’s experiences, namely IT specialists and supervisory staff. Multidisciplinary teams working on a common system proved useful in streamlining operations and formalizing processes. With the increasing digitization and availability digital tools, there is an increasing need for IT and public health personnel to speak the same language. Individuals at this nexus of public health and digital health literacy should be recognized as valuable assets for the public health workforce. Increased advocacy is needed to ensure minimum workforce requirements are met, including balanced teams to achieve collective competence across the epidemiology workforce [44].

This study emphasized persistent challenges with large data volumes witnessed during the COVID-19 pandemic. Fortunately, specific feature requests on loading time, pagination, filters and time localization were addressed in subsequent Go.Data versions [45]. The COVID-19 pandemic was an important opportunity to expand the tool beyond its original development use for smaller focal outbreaks, and re-iterated the importance of adapting based on evolving needs of the outbreak response landscape. Given high value placed on dialogue with WHO support team and across users, collecting and addressing feedback in a timely fashion should be among key priorities for WHO and GOARN partners.

### Strengths and limitations

A major strength of this study was its coverage and representation across all WHO regions. Regional distribution of participants roughly mirrored that of overall Go.Data implementations during COVID-19, and included participant viewpoints across multiple languages and diverse institutions. However, this study also had several limitations. Given that Go.Data installation files are freely shared by WHO and GOARN partners across headquarters, regional and country offices, the team did not have full visibility of every historical and existing implementation, and only knows of institutions that reach out directly for support. Due to this, the research team likely missed implementations when undertaking participant recruitment. The research team sought to remedy this by engaging WHO regional and country offices to follow up on leads if contact information was missing or not clear, prior to screening the implementation database. As is often the case with qualitative methods, there is the risk that both the interviewers and interviewees were exposed to bias. The research team sought to control for this bias via trainings on interviewing techniques and extensive piloting of the study instruments. In addition, the research team cannot guarantee completely the quality of the survey data as we are not sure if the right focal person completed it. For the scope of this study, the research team assumed that all survey responses were filled appropriately and used the responses accordingly.

## Conclusion

This study contributes to improved transparency on Go.Data’s global use during COVID-19 and provides steer for where WHO and GOARN partners should target future support. Study findings overall emphasized that Go.Data is not a “silver bullet” solution and relies on a capacitated and well-supervised team, with minimum IT infrastructure in place, in order to work as intended. Although the tool has limitations, Go.Data’s track record in accessibility and adaptability can be a foundation to build on as WHO’s continues development and endorsement of standards-based tools during outbreaks. Concerted preparedness efforts across the domains of workforce composition, data architecture and political sensitization should be prioritized as key ingredients for any successful Go.Data implementation, including increased digital literacy across the public health workforce. Continued dialogue between WHO and implementors, including via forums for countries to share experiences, will ensure the tool and support are reactive to evolving user needs.

## Data Availability

The data collection instruments and data are available from the corresponding author upon request.

## Abbreviations

COVID-19: Coronavirus disease 2019
FETP: Field Epidemiology Training Programs
GOARN: Global Outbreak Alert and Response Network
iOS: IDevice Operating System
IDI: In-depth interview
IT: Information technology
OSS: Open source software
WHO: World Health Organization
UN: United Nations
